# A Study on Drilling of CFRP/Ti Stacks: Temperature Field and Thermal Damage of the Interface Region

**DOI:** 10.3390/ma16072586

**Published:** 2023-03-24

**Authors:** Chen Chen, Aixu Wang, Zhi Zheng, Qing Zhao, Zhanli Shi, Yongjie Bao

**Affiliations:** 1 Naval Architecture and Ocean Engineering College, Dalian Maritime University, Dalian 116026, China; 2 School of Mechanical Engineering, Dalian University of Technology, Dalian 116024, China; 3 Marine Engineering College, Dalian Maritime University, Dalian 116026, China

**Keywords:** CFRP/Ti stacks, drilling, temperature field, interface region, thermal damage

## Abstract

Carbon fiber reinforced plastics (CFRP)/titanium alloy (Ti) stacks have been widely used in aviation field due to the superior mechanical properties. During integrated drilling of CFRP/Ti stacks, serious damage occurs in the CFRP layer because of the disparate properties of two stack components. Heat accumulation and thermal induced damage are typical and critical issue during drilling stacks, especially in the interface region. In this study, in order to deeply analyze the thermal influence of the interface region, a numerical model based on the finite difference method is developed to predict the three-dimensional drilling temperature field. Experiments with accurate measurement point are conducted to valid the rational of temperature prediction model. The results confirm that the temperature distributions predicted by numerical study have good agreements with the experimental results and the maximum error is about 10.3%. Furtherly, based on the drilling experiments, it can be found that thermal damage induced by cutting heat occurs as discoloration rings around the hole which could cause the elastic modulus of resin matrix decrease. An empirical model of thermal damage with maximum drilling temperature of the interface region are developed with the correlation of R^2^ = 0.97. The findings point out that as the maximum drilling temperature exceeds 410 °C, serious thermal damage could occur in the resin matrix of CFRP layer.

## 1. Introduction 

Carbon fiber reinforced plastics (CFRP) are widely used in aviation and aerospace industries due to the superior characteristics such as high specific strength, high specific stiffness and design flexibility [1,2,3]. Working as structure components, CFRP plates are usually connected and assembled with titanium alloy plates forming as CFRP/Ti stacks. For these stack structures, bolting and riveting are common methods for assembling, which requires huge amount of drilling operations [4]. According to statistics, more than 60% of the scrapped parts in the assembly process are caused by unqualified holes [5]. 

In actual holes machining process, CFRP/Ti stacks are drilled together to meet the machining accuracy and machining efficiency instead of drilling CFRP and Ti layer separately. However, the integrated drilling of CFRP/Ti stacks is a challenging task due to the discrepant machining properties of CFRP and Ti [6,7].

In CFRP/Ti stacks drilling process, serious mechanical damages occur in the CFRP layer such as delamination, tearing, fiber pullout. In order to reduce the aforementioned machining damage, many analytical researches and experimental studies have been carried out to achieving some favorable results [8,9,10]. However, to obtain high quality holes, related researches should not only deal with the mechanical damage, but also focus on the thermal damage caused by cutting heat. The glass transition temperature of CFRP is approximately to 180 °C, over which, the resin matrix degradation occurs within the subsurface layers [11,12]. Moreover, as drilling of CFRP/Ti stacks, huge amount of cutting heat is generated by drilling of Ti layer and cannot be dissipated effectively, which could induce local heat accumulation in the interface region of the stacks. Furtherly, the accumulated cutting heat will aggravate the degradation of resin matrix, which could reduce the strength of the materials and exacerbate the machining damage [13]. 

To reveal the phenomenon and the formation of thermal damage, some drilling experiments of stacks are conducted in the open literatures. Ramulu [14] found the discoloration rings occurred around the hole edge of the composite layer, which was induced by the cutting heat during drilling of Gi/Bi and Ti stacks. Kim [15] compared the hole qualities of CFRP layer during drilling of CFRP/Ti stacks and CFRP singly, and reveal that thermal damage occurs as drilling of stacks due to the cutting heat generated by drilling Ti layer. The aforementioned researches confirmed that thermal damage of composite is more serious in drilling of stacks and cutting heat is direct factor affecting the thermal damage. 

Although some techniques for stacks such as MQL and CO_2_-assisted drilling can reduce the drilling temperature and thermal damage [16], the formation reasons for thermal damage still need to be discussed. As an important indicator of cutting heat, drilling temperature can reflect the thermal state of cutting region effectively. Therefore, in order to analyze the heat affected damage deeply, many scholars focus on the drilling temperature during drilling of stacks first. Many temperature measurement methods are developed to acquire the drilling temperature online, among of these, the method of embedding thermocouples into workpieces or drill bits are the most-used method to capture the cutting temperature in the drilling process, especially for the invisible regions such as cutting region and interface region of the stacks. Brinksmeie [17] measured the drilling temperature of Al/CFRP/Ti stacks, and found that the drilling temperature reached the peak value as drilling of Ti layer and drilling temperature of Ti layer was far higher than that of CFRP layer. Wang [18] developed a rotating measurement system using thermocouples embedded in the drill bit to measure the drilling temperature during drilling of CFRP/Al stacks. The results confirmed that drilling temperature of the interface region is higher than the glass transition temperature of resin matrix, which could cause thermal damage of composite to be occurred in this region. 

Apart from the experimental measured methods, temperature field prediction during the drilling process were studied by several scholars. Liu [19] simplify the heat source during helix milling CFRP/Ti stacks as two parts: the arc heat source generated by the side edge of the tool and the linear heat source generated by the bottom edge, and established a non-steady heat conduction model with the anisotropy of composite to obtain the temperature distribution in machining process. Xu [20,21] proposed that the heat source generated from the friction between tool flank face and the workpieces, and developed an analytical model to predict the temperature rise for CFRP drilling. Montoya [22] decomposed the heat generated by drilling process as cutting heat located on the cutting edge and friction heat located on the drill margins. And a numerical model was developed to obtain the temperature field distribution of CFRP/Al stacks drilling process. 

However, as drilling of stacks, heat transfer is complex due to the discontinuous heat conduction in the interface region of the stacks. The existing researches on the drilling temperature, especially in the interface region is still insufficient. The previous study [12] confirmed that thermal damage induced by the cutting heat occurs around the hole of composite layer during drilling of stacks. The relations between thermal damage and drilling temperature is not clear. 

For this reason, in this manuscript, numerical models based on the finite difference method are developed to predict drilling temperature with the discontinuity of thermal conduction in the interface region of stacks. Experiments with accurate location of measure point are conducted to verify the assumption of analytical model and the values of drilling temperature in the interface region. Based on the drilling experiments, the morphology of thermal damage induced by cutting heat are obtained, and the relation between drilling temperature and thermal damage are deduced. This research can be a better guidance in predicting the cutting temperature during drilling of stacks and suppressing the thermal damage induced by cutting heat.

## 2. Materials and Methods

### 2.1. Modeling of the Drilling Temperature Field

#### 2.1.1. Modeling of the Heat Source

**Figure 1:**
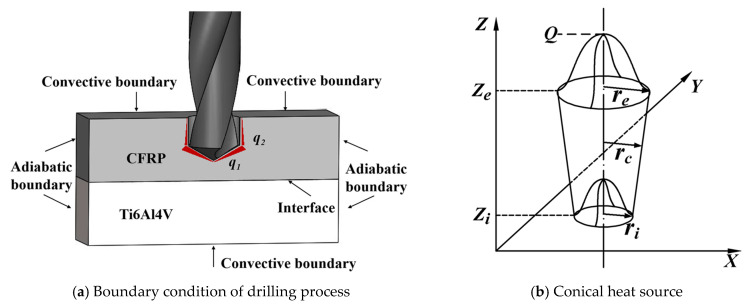
Boundary condition and heat source of drilling process.

The materials are removed by the chisel edge and main cutting edges of the twist drill, and the minor cutting edges only play guiding roles in the drilling process. Hence, in this model, the heat is mainly generated by materials removed, which distributes on chisel edge and main cutting edges, as *q*_1_ shown in Figure 1a. On the contrary, the heat generated by minor cutting edges *q*_2_ is little and can be neglected in calculation model. 

In the drilling process, heat distribution along the tool radius meet the Gaussian attenuation, and the heat source generated by chisel edge and main cutting edges during drilling process is approximately simplified as a conical heat source moving downwards [23], as shown in Figure 1b.

The total energy for drilling process can be expressed as

(1) $$P=M\omega +F_{z}v_{f}$$

where, *ω* is the angular velocity of the drill, *v_f_* is the feed velocity, *M* and *F_z_* are the torque and thrust force. The above parameters are measured through experiment.

The energy converted to heat and transferred to the workpiece is expressed as

(2) $$Q_{0}=\eta P$$

where, *η* is the proportional of energy transformed to workpiece.

The superficial area of the heat source can be simplified as the surface of the cone which is same with the contact region between tool and workpiece. The heat flux *q_T_* on unit area of the conical surface can be calculated as Equations (3) and (4)

(3) $$q_{T}=\frac{9Q_{0}}{\pi (1-e^{-3})}\cdot \frac{1}{\left(z_{e}-z_{i})(r_{e}^{2}+r_{e}r_{i}-r_{i}^{2}\right)}\cdot \exp(-\frac{3(x^{2}+y^{2})}{r_{c}^{2}})$$

(4) $$r_{c}=f(z)=r_{i}+(r_{e}-r_{i})\frac{z-z_{i}}{z_{e}-z_{i}}$$

where, *r_e_* and *r_i_* are the maximum radius and minimum radius of the conical heat source respectively, *r_c_* is the parameter related to the height of the heat source, *z_e_* and *z_i_* are the maximum value and minimum value in height of the conical heat source respectively. 

#### 2.1.2. Establishment of the Differential Equation of Thermal Field Model

Heat transferred to the workpiece is continually accumulated in the drilling process. Therefore, the temperature field during drilling of stacks is a non-steady state. The equation of heat conduction during the drilling process can be expressed as follows

(5) $$k_{x}\frac{\partial^{2}T}{\partial x^{2}}+k_{y}\frac{\partial^{2}T}{\partial x^{2}}+k_{z}\frac{\partial^{2}T}{\partial x^{2}}+q_{T}(x,y,z)=\rho c\frac{\partial T}{\partial t}$$

where, *k_x_*, *k_y_*, *k_z_* are thermal conductivities of the workpieces in *X*, *Y*, *Z* directions respectively, *T* is the relative temperature variation, *ρ* is the density of the workpieces and *c* is the specific heat of workpieces. 

To solve the thermal differential equation, the geometric conditions, boundary conditions and initial conditions need to be determined. The size of workpiece of CFRP and Ti layer are defined as *l_x_*, *l_y_*, *l_zC_* and *l_x_*, *l_y_*, *l_zT_* respectively. The upper and lower surfaces in the heat conduction model are set as convective boundaries. The boundaries conditions are expressed as follows

(6) $$h\cdot T=k_{z}\frac{\partial T}{\partial z}{}_{}\left(z=0,z=l_{zC}+l_{zT}\right)$$

where, *h* is the coefficient of thermal conduction. 

The surrounding four surfaces are less affected by the heat source and can be assumed to be insulating surfaces. Hence, the temperature of the surrounding four surfaces are approximately to the ambient temperature. The boundary conditions of surrounding four surface are expressed as Equation (7).

(7) $$\begin{array}{l} \frac{\partial T}{\partial x}=0{}_{}\left(x=0,x=l_{x}\right)\\ \frac{\partial T}{\partial y}=0{}_{}\left(y=0,y=l_{y}\right) \end{array}$$

As the CFRP and Ti layer are connected in mechanical form and the surface of the interface of two materials cannot contact completely. Resistance of thermal conduction exist in the uncontacted part, which makes the thermal conduction discontinuous in the interface region. In this study, thermal conductivity coefficient of the interface region is proposed to describe the change of drilling temperature between the adjacent surfaces of two layers in the interface region. The thermal conduction of the interface region is expressed as

(8) $$T_{z=l_{z1}+\Delta z}=\varepsilon \cdot T_{z=l_{z1}}$$

where, *ε* is the coefficient of thermal conduction of the interface region. The value of *ε* is determined through the drilling experiment. $T_{z=l_{z1}}$ is the drilling temperature in the underside of the upper layer, $T_{z=l_{z1}+\Delta z}$ is the drilling temperature in top surface of the lower layer.

In order to calculate the thermal field, the drilling area is discretized by the grid area [24]. The step size of three directions of *X*, *Y*, *Z* are set as Δ*x*, Δ*y* and Δ*z* respectively, and the mesh number of three directions are expressed as *N_x_*, *N_y_* and *N_z_* respectively.

(9) $$\begin{array}{l} N_{x}=l_{x}/\Delta x\\ N_{y}=l_{y}/\Delta y\\ N_{z}=\left(l_{zC}+l_{zT}\right)/\Delta z \end{array}$$

Initial drilling temperature of discretization node of the temperature field model is expressed as follows

(10) $$T_{i,j,k}^{0}=T_{0}i\in \left[0,N_{x}\right];j\in \left[0,N_{y}\right];k\in \left[0,N_{z}\right]$$

where, *i*, *j*, *k* is the node in three directions of *X*, *Y*, *Z* respectively. *T*_0_ is the ambient temperature.

Based on the above theories, heat conduction equation can be written in difference forms as

(11) $$\begin{array}{l} k_{x}\frac{T_{i-1,j,k}^{n}+T_{i+1,j,k}^{n}-2T_{i,j,k}^{n}}{\Delta x^{2}}+k_{y}\frac{T_{i,j-1,k}^{n}+T_{i,j+1,k}^{n}-2T_{i,j,k}^{n}}{\Delta y^{2}}+k_{z}\frac{T_{i,j,k-1}^{n}+T_{i,j,k+1}^{n}-2T_{i,j,k}^{n}}{\Delta z^{2}}\\ +q_{T}\left(i\cdot \Delta x,j\cdot \Delta y,k\cdot \Delta z\right)=\rho c\frac{T_{i,j,k}^{n+1}-T_{i,j,k}^{n}}{\Delta t} \end{array}$$

The temperature field of drilling CFRP/Ti stacks can be obtained through the proposed numerical model by running the Microsoft C++ program.

### 2.2. Experimental Validation

Drilling experiments are conducted to verify the veracity of the drilling temperature preidiction model. The experiments are carried out on GONA CNC machining center with the workpiece held in a rigid fixture attached to the Kistler 9257B 3-component platform dynamometer. The sampling frequency of dynamometer is 20 kHz. All the experiments are performed at ambient temperature of 25 °C. 

**Figure 2:**
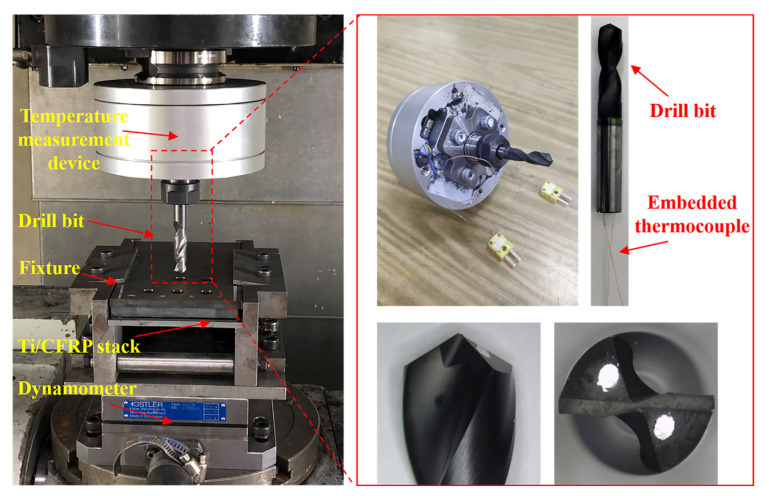
Experiment setup and drill bit.

**Figure 3:**
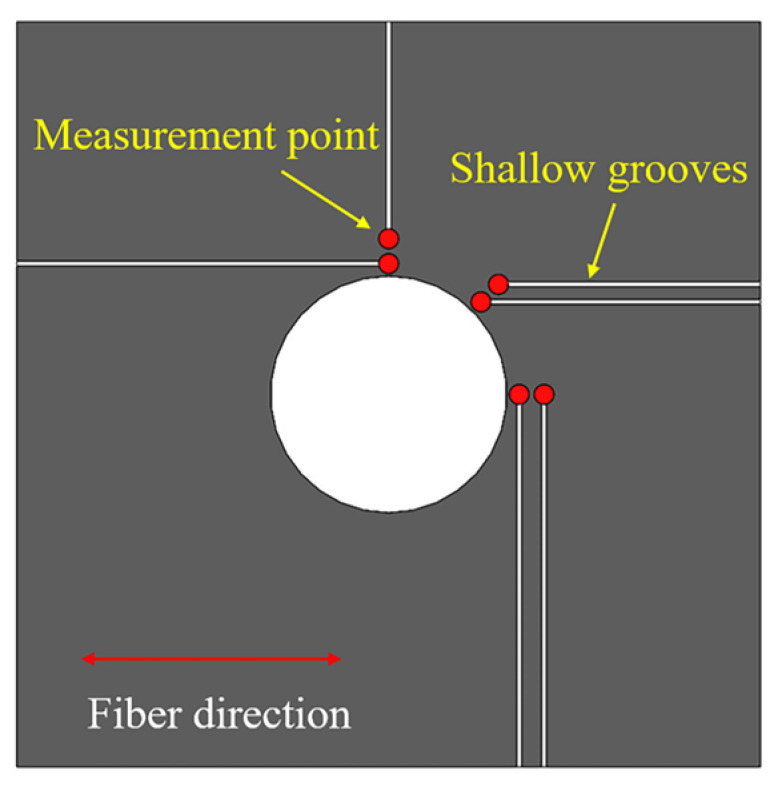
Locations of temperature measurement of interface region.

Thermocouples are used to measure the temperature in drilling process. A rotating temperature measuring system is developed to measure the drilling temperature of cutting region. K-type thermocouples are pre-embedded through the inner holes of the tool and fixed with thermocouple adhesive. The one endpoint of the thermocouple is contacted with the inside wall of the inner holes to keep the same positions of temperature measurements. The another endpoint of thermocouple is connected to the data acquisition module located in the inside of the handle. Meanwhile, in order to verify the calculation results of drilling temperature in the interface region, shallow grooves are process through laser processing method, where thermocouples are held and fixed in. The depth of the grooves is about 0.5 mm which is approximately to the diameter of endpoint of the thermocouples. Temperature of 6 positions are measured to verify the accuracy of the drilling temperature field prediction model, and the 6 positions are located in three directions of included angle of 0°, 45° and 90° to the fiber direction and the distance between the measurement points and hole edge are 0.5 mm and 1.5 mm respectively. Experimental setup and the location of the measurement position are shown in Figure 2 and Figure 3. 

The unidirectional T800/X850 CFRP laminate and Ti6Al4V titanium alloy are used in this study. The CFRP laminate with thickness of 8.0mm is made up of 40 unidirectional plies. The thickness of Ti is 6.4 mm. Detailed properties of CFRP and Ti are list in Table 1. 

The drill bits used in this study are YG8 cemented drills with diameter of 9.53 mm, point angle of 118° and diamond coating. Two inner holes are located in the flank surface of the drill, which are used for pre-embedded thermocouples. The morphology of the drill bit is shown in Figure 2 and the geometric parameters of drill bit are list in Table 2. Drilling experiments with same parameters are repeated 3 times using a new drill bit.

**Figure 4:**
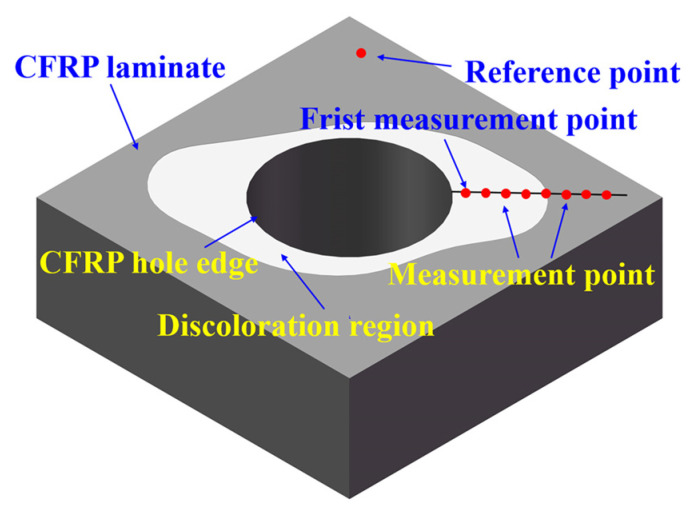
Schematic of the measurement points of resin matrix.

Besides, based on the previous study, after drilling process of CFRP/Ti stakcs, discoloration rings occur around the CFRP holes. Nanoindenter is used to measure the elastic modulus of resin matrix of discoloration region for the purpose of verifying the relation between discoloration region and thermal damage region. The 8 measurement positions are selected in same direction with interval distance of 500 μm and the distance between first measurement position and hole wall is 500 μm. The measurement positions span the discoloration region and non-discoloration region. Besides, a measurement position far away from the heat affection region is selected as a standard point. Schematic of the measurement points are shown as Figure 4. 

Thrust force, torque and drilling temperature are obtained through drilling experiment. The parameters for the temperature field model are the mean values of the experiments measured repeated for 3 times. The heat flux on unit area can be obtained based on the parameters experimental measured. The proportional coefficient of thermal conductivity of the interface region and proportional coefficient of energy transformed to heat are deduced based on the temperature measured from the experiments. Relevant parameters for temperature field prediction under the condition that stacking sequence is Ti to CFRP and drilling parameters are *n* = 600 rpm and *v_f_* = 0.03 mm/rev are shown in Table 3. 

## 3. Results and Discussion

### 3.1. Drilling Temperature of Cutting Region

**Figure 5:**
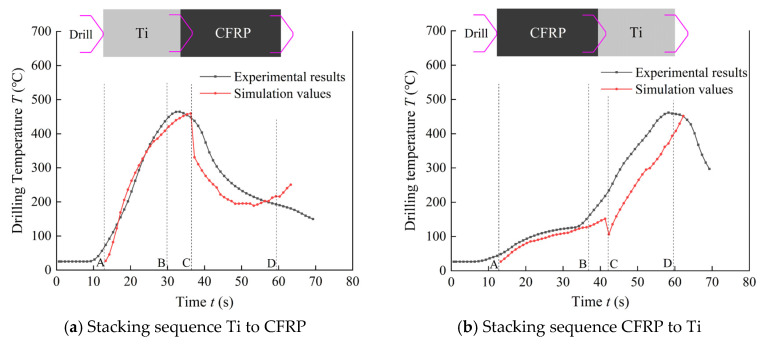
Drilling temperature of cutting region.

Based on the drilling temperature field prediction model and drilling experiments, drilling temperature of the cutting region predicted and experimental measured are obtained and shown in Figure 5. From Figure 5a, it can be inferred that as the stacking sequence is Ti to CFRP, the character of drilling temperature presents 3 stages. The drilling temperature increases rapidly as drilling of Ti layer and reaches the maximum value when drilling the interface region. As the drill moving downwards furtherly, drilling temperature of the cutting region decreases, due to the amount of Ti layer removed by the tool reduced and the amount of CFRP layer removed increased. The predicted drilling temperature are in good agreement with the experimental values in the temperature rising stage AB as shown in Figure 5a. The maximum error of drilling temperature in the cutting region is about 6.7%. As the drill moves downwards, the drilling temperature decreases sharply as the drill moving into CFRP layer. 

In this stage, the predicted drilling temperature is slightly lower than the experiment values because that the thermocouples are located on the flank surface of the drill, and the experimental measured values are drilling temperature of the tool which are influenced by the heat accumulated during drilling of the Ti layer. By contrast, in the temperature field prediction model, the locations of the predicted values are selected in the cutting region in the CFRP layer instead of the tool, and the heat accumulate would induce temperature rising in the CFRP layer. 

When the stacking sequence is CFRP to Ti, the predicted drilling temperature agrees well with the experimental values as drilling of CFRP layer and the interface region, as shown in Figure 5b. Drilling temperature in the upper surface of Ti layer decreases as drilling of the interface region due to the discontinuous contact between CFRP and Ti layer.

In drilling temperature prediction model, drilling temperature of the cutting zone are selected in the workpieces instead of the flank surface of the tool in drilling experiments. The drilling temperature in the stage of drilling in and drilling out can not be obtained through the drilling temperature prediction model. Hence, the total time of the drilling temperature curve are different in prediction model and drilling experiments. 

### 3.2. Drilling Temperature of Interface Region

As the drilling temperature with the stacking sequence of Ti to CFRP is much higher than that in the stacking sequence of CFRP to Ti, which could exceed the glass transition temperature of the resin matrix and cause serious thermal damage of resin, in this manuscript, drilling temperatures of interface region with stacking sequence of Ti to CFRP are chosen to verify the veracity of drilling prediction model furtherly. 

**Figure 6:**
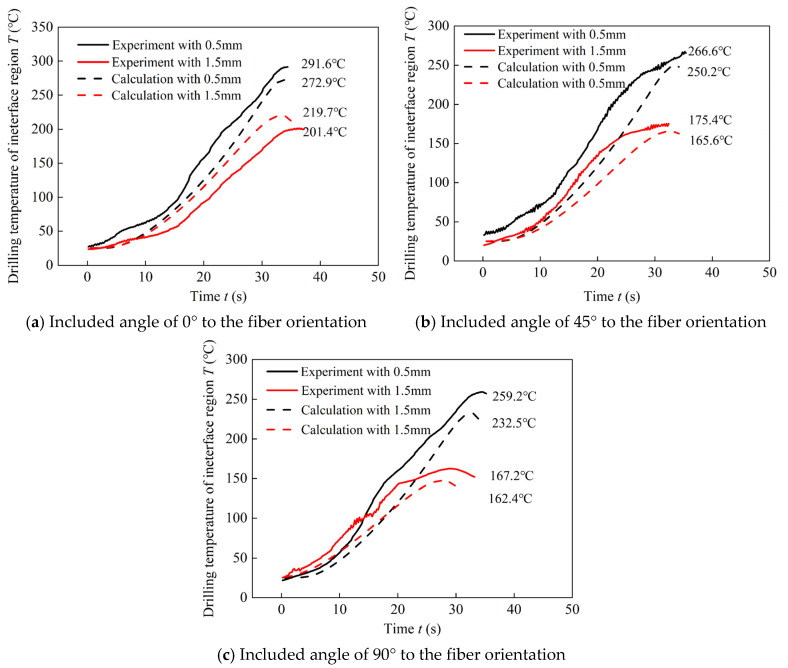
Comparation of drilling temperatures of interface region gained by prediction models and experimental test.

The drilling temperature of the interface region predicted and experimental measured of the 6 locations selected are obtained as shown in Figure 6. Based on the results, it can be learned that drilling temperature of predicted and experimental measured in 6 positions are in same trend respectively. Temperature of predicted values and experimental measured values of the selected position is about 10.3%. The analytical model can predict the drilling temperature of interface region effectively. Moreover, drilling temperature in different positions during drilling of stacks can be obtained through the drilling temperature prediction model.

**Figure 7:**
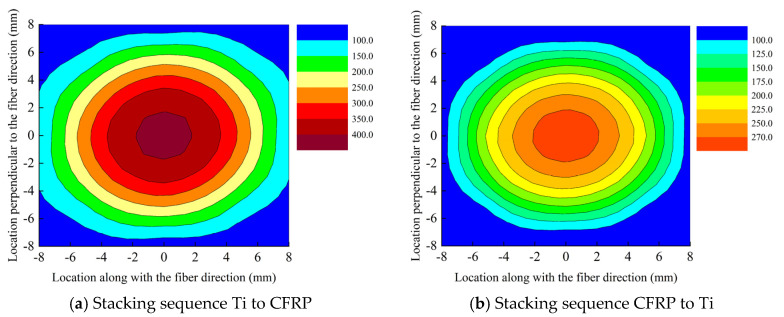
Drilling temperature field of interface region at drilling parameters of *n* = 600 rpm and *f* = 0.03 mm/rev.

Based on the developed model, drilling temperature field of the interface region are obtained. Figure 7 shows the drilling temperature field of interface region of two stacking sequences with drilling parameters of *n* = 600 rpm and *v_f_* = 0.03 mm/rev. Based on the results, it can be indicated that drilling temperature of the interface region with the stacking sequence of Ti to CFRP is much higher than that with stacking sequence of CFRP to Ti in same positions. Figure 7a shows that maximum drilling temperature at the position of 0.5 mm away from the hole edge in three directions of 0°, 45° and 90° to the fiber direction are 272.9 °C, 250.2 °C and 232.5 °C respectively, which are all above the glass transition temperature of the epoxy resin. Hence, severe thermal damage occurs in the epoxy resin near the hole side. Besides, drilling temperature at the position of 1.5 mm away from the hole edge along the fiber orientation is about 200 °C. By contrast, the maximum drilling temperature of the interface region at the positions of 0.5 mm away the hole edge in three directions of 0°, 45° and 90° to the fiber direction are 212.8 °C, 196.4 °C and 180.8 °C respectively, which are little higher than the glass transition temperature of the epoxy resin. Hence, thermal damage can also occur in this circumstance. While, temperature in the positions of 1.5 mm away from the hole edge are obvious lower than the glass transition temperature of the epoxy resin. 

In addition, it can be learned that the thermal field of CFRP layer in the interface region presents elliptical distribution. This is mainly because that the thermal conductivity of carbon fiber is higher than that of the resin matrix. Drilling temperature is much higher in the position along the fiber direction than that perpendicular to the fiber direction in the same distance. The shape of the thermal field of the interface region is distinct between two stacking sequence. When stacking sequence is Ti to CFRP, the interface region locates in the drilling-in of the CFRP layer. Huge amount of cutting heat is generated as drilling of Ti layer and transfers into CFRP layer through the tool, hence, the anisotropy of CFRP only has the influences on the thermal conductivity of CFRP. By contrast, when drilling from CFRP to Ti, the interface region locates in the drilling-out of the CFRP layer and heat source which affects the drilling temperature of interface region is generated by cutting CFRP. The anisotropy of CFRP has a huge influence on the heat generation and thermal conductivity which could cause the elliptical ratio of the thermal field with stacking sequence of CFRP to Ti is larger than that with stacking sequence of Ti to CFRP. 

### 3.3. Thermal Damage

**Figure 8:**
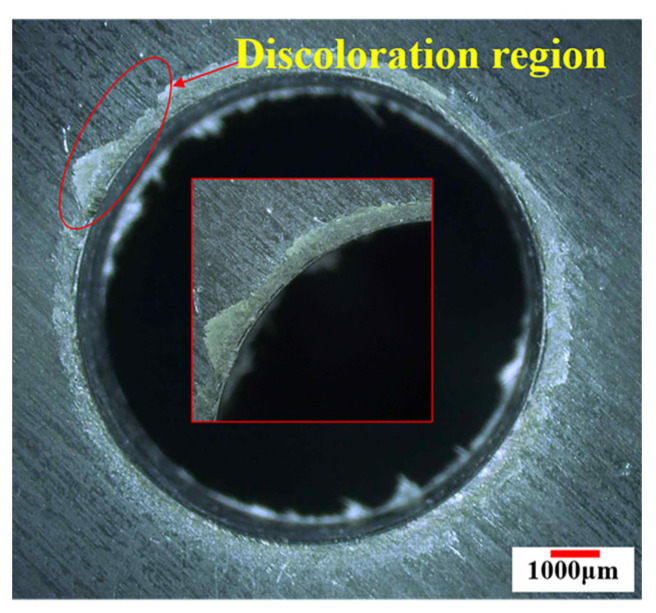
The morphology of thermal damage of CFRP in the interface region.

Figure 8 shows the morphology of CFRP holes in the interface region after drilling process. It can be found that discoloration rings induced by cutting heat occur around the hole in the interface region. 

**Figure 9:**
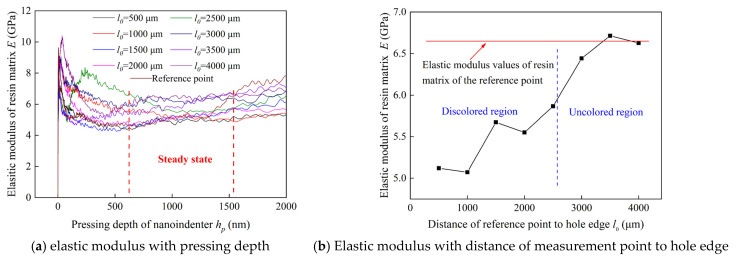
Elastic modulus of rein matrix experimental measured.

In order to verify the relationship between discoloration region and thermal damage region, elastic modulus of resin matrix of 8 selected measurement points as expressed in Section 2.2 are obtained and shown in Figure 9. Based on the results, it can be learned that the value of elastic modulus of resin matrix in the discoloration region of surface layer is lower than that of non-discoloration region. Besides, the values of elastic modulus of resin matrix increase as the distance between selected position and hole wall increases, and the value of resin matrix of non-discoloration region is approximate to that of uncut area. Hence, in this paper, it can be concluded that the thermal damage area of CFRP can be approximately determined based on the discoloration. 

Meanwhile, it can be found that there is a significant difference between thermal damage region and heat affect region which is determined by exceeding the glass transition temperature of the resin matrix. The area of heat affect region is larger than the thermal damage region. 

Furtherly, combined with the values of drilling temperature in the interface region, it can be inferred that resin matrix suffers the cutting heat could cause the drilling temperature exceed the glass transition temperature in a short time, whereas the material properties do not suffer a large extent decrease. Based on the above analysis, thermal damage of the CFRP is shown as the discoloration of resin matrix. Therefore, the region of thermal damage of CFRP can be determined by the discoloration region. In order to evaluate the thermal damage quantitatively, thermal damage factors are calculated based on the ratio of discoloration area to the hole area as proposed in the previous study [12].

Moreover, cutting heat can affect the thermal damage of CFRP directly, and drilling temperature is a key indicator of cutting heat in the cutting region. Therefore, establish a correlation model between drilling temperature and thermal damage factor can predict the influence of cutting heat on thermal damage of CFRP in the drilling process effectively.

**Figure 10:**
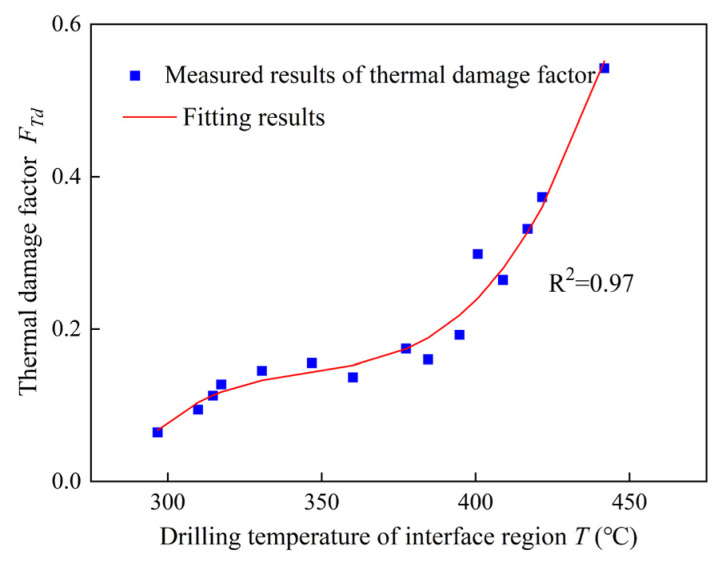
Thermal damage factor with drilling temperature.

In this study, combined with the maximum drilling temperatures of the interface region in drilling of stacks predicted through the drilling temperature field model and thermal damage factor of CFRP calculated, an empirical model of thermal damage factor with maximum drilling temperature is developed, as shown in Figure 10.

A good correlation of R^2^ = 0.97 is deduced from the curves, and the trend between thermal damage factor and maximum drilling temperature is fitted as Equation (12).

(12) $$F_{Td}=4\times 10^{-7}T^{3}-4.15\times 10^{-4}T^{2}+0.144T-16.604$$

where, *F_Td_* is the thermal damage factor, *T* is the maximum drilling temperature.

According to the relation between thermal damage factor and maximum drilling temperature, it can be learned that thermal damage factors increases steadily first and then increase sharply as the maximum drilling temperature increases. As the maximum drilling temperature exceeds 410 °C, serious thermal damage could occur in the resin matrix of CFRP layer.

## 4. Conclusions

In this study, a numerical model based on the finite difference method is developed for temperature field prediction during drilling of CFRP/Ti stacks. The drilling temperature field distribution of interface region and the thermal induced damage are obtained. According to the analysis of the calculation and experimental results, conclusions can be drawn as follows:

(1) The proposed numerical model based on the finite difference method can predict the drilling temperature of cutting region and interface region effectively, and the maximum error is about 10.3%.

(2) Elliptical shape temperature field occurs in the CFRP layer of the interface region. As the stacking sequence is Ti to CFRP, drilling temperature is higher than that with the stacking sequence of CFRP to Ti in same positions. Drilling temperature of the interface region could exceed 270 °C in the positon of 0.5 mm away from the hole edge under the drilling parameters of *n* = 600 rpm and *f* = 0.03 mm/rev, which could cause serious thermal damage of CFRP.

(3) Thermal damage occurs as discoloration rings around the hole where the elastic modulus of the resin matrix decreases observably. As drilling temperature exceeds about 410 °C, serious thermal damage could occur in the resin matrix of CFRP layer.

## Figures and Tables

**Table 1:** Thermophysical properties of unidirectional CFRP laminate and titanium alloy.

Workpiece Material	*c* (J/kg·K)	*ρ* (kg/m^3^)	*k_x_* (W/(mk))	*k_y_* (W/(mk))	*k_z_* (W/(mk))
T800/X850 CFRP	195	1530	4.6	0.78	0.78
Ti6Al4V	113	4430	7.0		

**Table 2:** Geometric parameters of the drill bit.

Diameter	9.53 mm
Cutting edge length	50 mm
Point angle	118°
Helix angle	25°
Coating	Diamond

**Table 3:** Parameters for temperature field of drilling CFRP/Ti stacks.

Parameter	Value
Thrust force of CFRP, *F_zC_* (N)	108.3
Torque of CFRP, *M_C_* (N·m)	0.65
Thrust force of Ti, *F_zT_* (N)	424.4
Torque of Ti, *M_T_* (N·m)	2.64
*v_f_* (mm/min)	18
*n* (r/min)	600
*h* (W·m^−2^·K^−1^)	20
*d* (mm)	9.53
*η*	0.17
*ε*	0.24
